# Knowledge, practice, and barriers to opportunistic salpingectomy during non-gynaecological surgery: international survey

**DOI:** 10.1093/bjsopen/zrag145

**Published:** 2026-09-28

**Authors:** Jeremy Meyer, Manuela Undurraga, Frédéric Ris, Thibaud Koessler, Intidhar Labidi-Galy, the TUBE Study Group, Francesco Abboretti, Ahmed Abdelsamad, Michel Adamina, Alberto Aiolfi, Vusal Aliyev, Elissavet Anestiadou, Alazar Berhe Aregawi, Claudia Armellin, Andrea Barberis, Daniel Benamran, Stephan Bischofberger, Luca Domenico Bonomo, Giuseppe Brisinda, Maria Bubenova, Nicolas Buchs, Pamela Buchwald, Giacomo Calini, Rory Callan, Valentin Calu, Fabio Carbone, Marco Catarci, Alexandros Chamzin, Luca Cigagna, Diego Coletta, Renan Carlo Colombari, Nicola Colucci, Rogier Crolla, Justin Davies, Aeris Jane Dublado, Lorenzo Epis, Francesco Ferrara, Tahar Fillali, Christina Fleming, Gaetano Florio, Pietro Fransvea, Zoe Garoufalia, Georgios Geropoulos, Eleftherios Gialamas, Guillaume Giudicelli, Ugo Giustizieri, Carmen Loredana Gorgan, Gianpiero Gravante, Argyrios Ioannidis, Orestis Ioannidis, Arda Isik, Ioannis Katsaros, Deborah Keller, Raja Basit N Khan, Niels Komen, Dimitris Korkolis, Bojan Kovacevic, Joël L Lavanchy, Jeroen Leijtens, Emilie Liot, Alexis Litchinko, Andrea Pierre Luzzi, Jin Jiun Mah, Kashish Malhotra, Draga-Maria Mandi, Angelo Alessandro Marra, Valentina Miacci, Pamela Milito, Ana Minaya Bravo, Blanca Montcusí, Dimitrios Moris, Adam Mylonakis, Peter Neary, Harish Neelamraju Lakshmi, Ionut Negoi, Antonella Nicotera, Aristotelis Nikitaras, Nuri Okkabaz, Lorenzo A Orci, Paolo Ossola, Livia Palmieri, Giulia Paradiso, Francesco Pata, Georgios Peros, Lorenzo Petagna, Thalia Petropoulou, Gopi Ramu, Stefano Restaino, Raffaello Roesel, Fausto Rosa, Fabian Rössler, Jacques-Emmanuel Saadoun, Kapil Sahnan, Ville Sallinen, Matteo Santoliquido, Giulio Aniello Santoro, Carlo Alberto Schena, Boris Schiltz, Dimitrios Schizas, Michele Scopelliti, Mostafa Shalaby, Marwan-Julien Sleiman, Amine Souadka, Moritz Benjamin Sparn, George Stavrou, Giovanni Tebala, Pierre-Alain Tokoto, Giovanni Tomasicchio, Charles Van Praet, Gabrielle H van Ramshorst, Carsten T Viehl, Danilo Vinci, Giuseppe Vizzielli, Lukas Werner Widmer, Muhammad Umar Younis, Matteo Zanello

**Affiliations:** Division of Digestive Surgery, Geneva University Hospitals, Geneva, Switzerland; Faculty of Medicine, University of Geneva, Geneva, Switzerland; Colorectal Cancer Center, Geneva University Hospitals, Geneva, Switzerland; Faculty of Medicine, University of Geneva, Geneva, Switzerland; Division of Gynaecology, Geneva University Hospitals, Geneva, Switzerland; Department of Oncology, Geneva University Hospitals, Geneva, Switzerland; Division of Digestive Surgery, Geneva University Hospitals, Geneva, Switzerland; Faculty of Medicine, University of Geneva, Geneva, Switzerland; Colorectal Cancer Center, Geneva University Hospitals, Geneva, Switzerland; Faculty of Medicine, University of Geneva, Geneva, Switzerland; Colorectal Cancer Center, Geneva University Hospitals, Geneva, Switzerland; Department of Oncology, Geneva University Hospitals, Geneva, Switzerland; Center of Translational Research in Oncohematology, Faculty of Medicine, University of Geneva, Geneva, Switzerland; Faculty of Medicine, University of Geneva, Geneva, Switzerland; Department of Oncology, Geneva University Hospitals, Geneva, Switzerland; Center of Translational Research in Oncohematology, Faculty of Medicine, University of Geneva, Geneva, Switzerland

Ovarian cancer causes over 200 000 deaths annually and its prognosis remains poor because it is usually diagnosed at an advanced stage^1^. Recognition that most high-grade serous carcinomas (HGSCs) originate in the fallopian tube has transformed ovarian cancer prevention strategies^2^. Major gynaecological societies now endorse opportunistic salpingectomy during elective surgery as a low-risk strategy to reduce future ovarian cancer risk^3^. In a commentary to these guidelines, the authors called for extension of these recommendations to non-gynaecological abdominal surgery. Non-gynaecological abdominal procedures are frequently performed in women aged over 40 years and provide direct intraoperative access to the adnexa, yet opportunistic salpingectomy is rarely performed in this context^4^. The TUBE (Tubal management Under Broad surgical Environments) study characterized current practices, knowledge, and barriers among abdominal surgeons.

Between April and May 2026, a cross-sectional survey was distributed electronically via REDCap tools hosted at the Geneva University Hospitals to abdominal surgeons internationally through surgical society mailing lists, professional networks, and direct personal contacts across Europe and beyond. Distribution was indirect through network contacts and society channels. Duplicate responses were prevented by REDCap session control. The questionnaire covered adnexal management practices, knowledge of HGSC pathogenesis, awareness of gynaecological guidance, and intention to change practice following a standardized educational prompt. Barriers were assessed using a five-point Likert scale (1, strongly agree; 5, strongly disagree).

Of 307 respondents, 277 completed the survey fully (median age 44 years, 81% men) (*Table S1*). The specialty distribution was general surgery 44%, gastrointestinal/ hepatopancreatobiliary surgery 49%, and urology 5%. Most respondents worked in academic public hospitals (55%) and performed over 100 abdominal procedures annually (59%).

Knowledge of HGSC pathogenesis was limited across all groups (*Table 1* and *Table S2*). Only 37% correctly identified the fallopian tube as the primary site of HGSC origin, and 41% recognized that opportunistic salpingectomy reduces ovarian cancer risk. Only 8% were familiar with existing gynaecological guidelines. Most surgeons (63%) had never performed elective salpingectomy opportunistically, and only 20% had ever offered risk-reduction adnexal removal to a patient. Following a standardized educational prompt, 53% expressed a willingness to discuss salpingectomy with eligible patients. Most respondents (87%) would consider salpingectomy if specialty-specific guidance were available. Lack of consent clarity (65%) and insufficient epidemiological evidence in abdominal surgery populations (61%) were the most frequently cited barriers (*Table 1* and *Table S3*).

**Table 1:** Knowledge and barriers by surgical specialty group

	General surgery (*n* = 121)	GI/HPB (*n* = 136)	Urology (*n* = 13)
**Knowledge (% correct responses)**			
Fallopian tube as primary site of HGSC origin	33%	40%	39%
Salpingectomy reduces ovarian cancer risk	38%	46%	31%
**Barriers (% agree or strongly agree)**			
Would consider if specialty-specific guidance existed	88%	86%	85%
Lack of consent clarity	66%	69%	54%
Insufficient epidemiological evidence	60%	65%	54%

Urology results should be interpreted with caution (13 respondents). GI/HPB, gastrointestinal and hepatopancreatobiliary (combining lower gastrointestinal, upper gastrointestinal, and hepatopancreatobiliary subspecialties); HGSC, high-grade serous carcinoma.

These findings reveal a critical knowledge gap across non-gynaecological surgical specialties regarding HGSC pathogenesis and the preventive potential of salpingectomy. The principal barriers appear to be structural rather than attitudinal. The absence of specialty-specific guidelines and clear consent frameworks, rather than reluctance among surgeons, is the major obstacle to implementation. Nearly one in four women diagnosed with HGSC has previously undergone abdominal or pelvic surgery during which salpingectomy was not performed, representing a quantifiable missed opportunity for prevention^5^. Development of abdominal surgery-specific recommendations, building on the recent European Society of Gynaecological Oncology consensus^3^, represents the most actionable intervention identified by this survey.

## Collaborators

Members of the TUBE Study Group: Francesco Abboretti (Lausanne University Hospital (CHUV), Lausanne, Switzerland); Ahmed Abdelsamad (Department of Surgery, University of Witten/Herdecke, Witten, Germany); Michel Adamina (Inselspital Bern University Hospital, Bern, Switzerland); Alberto Aiolfi (IRCCS Ospedale Galeazzi-Sant'Ambrogio, Milan, Italy); Vusal Aliyev (Department of General Surgery, Alibey Hospital, Istanbul, Turkey); Elissavet Anestiadou (4th Department of General Surgery, General Hospital of Thessaloniki ‘G. Papanikolaou’, Aristotle University of Thessaloniki, Thessaloniki, Greece); Alazar Berhe Aregawi (Hawassa University College of Medicine and Health Sciences, Hawassa, Ethiopia); Claudia Armellin (Policlinico di Abano Terme, Abano Terme, Italy); Andrea Barberis (E.O. Ospedali Galliera, Genoa, Italy); Daniel Benamran (Division of Urology, Geneva University Hospitals, Geneva, Switzerland); Stephan Bischofberger (HOCH Kantonsspital St. Gallen, St. Gallen, Switzerland); Luca Domenico Bonomo (Rivoli Hospital, Rivoli (TO), Italy); Giuseppe Brisinda (Fondazione Policlinico Universitario A. Gemelli IRCCS, Rome, Italy); Maria Bubenova (Landesklinikum Waidhofen an der Ybbs, Austria, Austria); Nicolas Buchs (Hôpital de La Tour, Geneva, Switzerland); Pamela Buchwald (Skåne University Hospital Malmö, Lund University, Malmö, Sweden); Giacomo Calini (Department of Medical and Surgical Sciences, Alma Mater Studiorum – University of Bologna; IRCCS Azienda Ospedaliero-Universitaria di Bologna, Bologna, Italy); Rory Callan (St Mark's Hospital, London, United Kingdom); Valentin Calu (University of Medicine and Pharmacy Carol Davila, Bucharest, Romania); Fabio Carbone (Istituto Nazionale Tumori IRCCS – Fondazione G. Pascale, Naples, Italy); Marco Catarci (Sandro Pertini Hospital, Rome, Italy); Alexandros Chamzin (First Propaedeutic Department of Surgery, Hippocration General Hospital of Athens, Athens, Greece); Luca Cigagna (CHU de La Réunion, Saint-Denis, France); Diego Coletta (Digestive and HPB Surgery Unit, IRCCS Regina Elena National Cancer Institute, Rome, Italy); Renan Carlo Colombari (Gregorio Marañón University Hospital, Madrid, Spain); Nicola Colucci (Department of Surgery, University of British Columbia, Vancouver, Canada); Rogier Crolla (Netherlands, Netherlands); Justin Davies (Cambridge University Hospitals NHS Foundation Trust, Cambridge, United Kingdom); Aeris Jane Dublado (Allied Care Experts (ACE) Medical Center Tacloban, Tacloban, Philippines); Lorenzo Epis (DISC – Università degli Studi di Genova, Genoa, Italy); Francesco Ferrara (University of Palermo, Palermo, Italy); Tahar Fillali (Department of Surgery, Béni Messous University Hospital, Algiers, Algeria); Christina Fleming (University Hospital Limerick, School of Medicine, University of Limerick, Limerick, Ireland); Gaetano Florio (ASL Roma 5, Rome, Italy); Pietro Fransvea (Fondazione Policlinico Universitario A. Gemelli IRCCS, Rome, Italy); Zoe Garoufalia (6th Surgical Department, Hygeia Hospital, Athens, Greece); Georgios Geropoulos (Watford General Hospital, Watford, United Kingdom); Eleftherios Gialamas (Geneva University Hospitals, Geneva, Switzerland); Guillaume Giudicelli (Division of Visceral Surgery, Geneva University Hospitals, Geneva, Switzerland); Ugo Giustizieri (Ospedale Vito Fazzi, Lecce, Italy); Carmen Loredana Gorgan (Regina Maria Hospital, Bucharest, Romania); Gianpiero Gravante (Azienda Sanitaria Locale (ASL) Lecce, Lecce, Italy); Argyrios Ioannidis (Athens Medical Center, Athens, Greece); Orestis Ioannidis (4th Department of Surgery, Medical School, Aristotle University of Thessaloniki, Thessaloniki, Greece); Arda Isik (Istanbul Medeniyet University, Istanbul, Turkey); Ioannis Katsaros (National and Kapodistrian University of Athens, Laikon General Hospital, Athens, Greece); Deborah Keller (Mayo Clinic, Phoenix, AZ, United States); Raja Basit N. Khan (Airedale NHS Foundation Trust, Keighley, United Kingdom); Niels Komen (University Hospital Antwerp, Antwerp, Belgium); Dimitris Korkolis (Department of Surgery, General Anticancer Oncology Hospital of Athens ‘Saint Savvas’, Athens, Greece); Bojan Kovacevic (UMC Zvezdara, Belgrade, Serbia); Joël L. Lavanchy (Clarunis – University Digestive Health Care Center, Basel, Switzerland); Jeroen Leijtens (Laurentius Ziekenhuis, Roermond, Netherlands); Emilie Liot (Geneva University Hospitals, Geneva, Switzerland); Alexis Litchinko (Department of Surgery, Bordeaux Colorectal Academy, Bordeaux, France); Andrea Pierre Luzzi (ASST Santi Paolo e Carlo, Milan, Italy); Jin Jiun Mah (Queen Elizabeth Hospital, Kota Kinabalu, Malaysia); Kashish Malhotra (Department of Surgery, Rama Medical College Hospital and Research Centre, Kanpur, India); Draga-Maria Mandi (Coltea Clinical Hospital, Bucharest, Romania); Angelo Alessandro Marra (Isola Tiberina – Gemelli Isola Hospital, Rome, Italy); Valentina Miacci (Campus Bio-Medico, Rome, Italy); Pamela Milito (IRCCS Policlinico San Donato, San Donato Milanese, Italy); Ana Minaya Bravo (Hospital Universitario del Henares, Madrid, Spain); Blanca Montcusí (Section of Colon and Rectal Surgery, Department of Surgery, Hospital del Mar, Barcelona, Spain); Dimitrios Moris (Department of Surgery, MedStar Georgetown University Hospital, Washington, DC, United States); Adam Mylonakis (First Department of Surgery, National and Kapodistrian University of Athens, Laiko General Hospital, Athens, Greece); Peter Neary (University Hospital Waterford, University College Cork, Cork, Ireland); Harish Neelamraju Lakshmi (Department of Surgical Oncology, Continental Hospitals, Hyderabad, India); Ionut Negoi (Carol Davila University of Medicine and Pharmacy; Clinical Emergency Hospital of Bucharest, Bucharest, Romania); Antonella Nicotera (Rivoli Hospital ASL TO3, Rivoli, Italy); Aristotelis Nikitaras (Athens Euroclinic, Athens, Greece); Nuri Okkabaz (Istanbul Atlas University, Istanbul, Turkey); Lorenzo A. Orci (Clinique Générale-Beaulieu, Geneva, Switzerland); Paolo Ossola (General Surgery 2U – Liver Transplant Centre, A.O.U. Città della Salute e della Scienza, Turin, Italy); Livia Palmieri (Ospedale Madre Giuseppina Vannini, Rome, Italy); Giulia Paradiso (Ospedale M.G. Vannini, Rome, Italy); Francesco Pata (Department of Pharmacy, Health and Nutritional Sciences, University of Calabria, Rende, Italy); Georgios Peros (Chirurgie Zentrum St. Anna, Lucerne, Switzerland); Lorenzo Petagna (AOU Pisana, Pisa, Italy); Thalia Petropoulou (Aretaieio University Hospital, Athens, Greece); Gopi Ramu (Department of Surgery, Chengelpet Medical College and Hospital, Chengelpet, India); Stefano Restaino (Azienda Sanitaria Universitaria Friuli Centrale, Udine, Italy); Raffaello Roesel (EOC Regional Hospital of Lugano, Lugano, Switzerland); Fausto Rosa (Department of Surgery, Università Cattolica del Sacro Cuore; Fondazione Policlinico Universitario Agostino Gemelli IRCCS, Rome, Italy); Fabian Rössler (Department of Surgery and Transplantation, University Hospital Zurich, Zurich, Switzerland); Jacques-Emmanuel Saadoun (Department of Surgical Oncology, Institut Paoli-Calmettes, Marseille, France); Kapil Sahnan (St Mark's Hospital, London, United Kingdom); Ville Sallinen (Helsinki University Hospital and University of Helsinki, Helsinki, Finland); Matteo Santoliquido (IRCCS Ospedale Policlinico San Martino, Genoa, Italy); Giulio Aniello Santoro (Humanitas Gavazzeni Hospital, Bergamo, Italy); Carlo Alberto Schena (Fondazione Poliambulanza Istituto Ospedaliero, Brescia, Italy); Boris Schiltz (Centre Hospitalier de Bienne, Biel/Bienne, Switzerland); Dimitrios Schizas (First Department of Surgery, National and Kapodistrian University of Athens, Athens, Greece); Michele Scopelliti (Chirurgia 2, ULSS2 Marca Trevigiana, Treviso, Italy); Mostafa Shalaby (Department of General Surgery, Mansoura University, Mansoura, Egypt); Marwan-Julien Sleiman (Geneva University Hospitals, Geneva, Switzerland); Amine Souadka (Department of Surgical Oncology, National Institute of Oncology, Mohammed V University, Rabat, Morocco); Moritz Benjamin Sparn (HOCH Health Ostschweiz Kantonsspital St. Gallen, St. Gallen, Switzerland); George Stavrou (417 NIMTS Hospital, Athens, Greece); Giovanni Tebala (Department of Digestive and Emergency Surgery, Azienda Ospedaliera S. Maria, Terni, Italy); Pierre-Alain Tokoto (Geneva University Hospitals, Geneva, Switzerland); Giovanni Tomasicchio (Azienda Consorziale Policlinico di Bari, Bari, Italy); Charles Van Praet (Department of Urology, Ghent University Hospital, Ghent, Belgium); Gabrielle H. van Ramshorst (Ghent University Hospital, Ghent University, Ghent, Belgium); Carsten T. Viehl (Centre Hospitalier de Bienne, Biel/Bienne, Switzerland); Danilo Vinci (Hospital del Mar, Barcelona, Spain); Giuseppe Vizzielli (University of Udine, Udine, Italy); Lukas Werner Widmer (Department of Surgery, Cantonal Hospital of Fribourg; University of Fribourg, Fribourg, Switzerland); Muhammad Umar Younis (Mediclinic City Hospital Dubai, Dubai, United Arab Emirates); Matteo Zanello (Department of General Surgery, IRCCS Azienda Ospedaliero-Universitaria di Bologna, Maggiore Hospital, Bologna, Italy).

## Supplementary Material

zrag145_Supplementary_Data

## Data Availability

Data are available from the authors upon reasonable request.
