# Supplementary material for: Knowledge, practice, and barriers to opportunistic salpingectomy during non-gynaecological surgery: international survey
Source: BJS Open. 2026 Sep 28;10(5):zrag145. doi: 10.1093/bjsopen/zrag145 (PMC13617668; doi:10.1093/bjsopen/zrag145)
Supplement: Supplementary file 1 [file zrag145_supplementary_data.docx]

**Knowledge, practice and barriers to opportunistic salpingectomy during non-gynaecological surgery: international survey**

Jeremy Meyer ^1,2,3^, Manuela Undurraga ^2,4,5^, Frédéric Ris ^1,2,3^, Thibaud Koessler ^2,3,5,6^, Intidhar Labidi-Galy ^2,5,6^, on behalf of the TUBE Study Group*

^1 Division of Digestive Surgery, Geneva University Hospitals, Switzerland^

^2 Faculty of Medicine, University of Geneva, Switzerland^

^3 Colorectal Cancer Center, Geneva University Hospitals, Switzerland^

^4 Division of Gynaecology, Geneva University Hospitals, Switzerland^

^5 Department of Oncology, Geneva University Hospitals, Switzerland^

^6 Center of Translational Research in Oncohematology, Faculty of Medicine, University of Geneva, Switzerland^

**Corresponding author.** Jeremy Meyer, Division of Digestive Surgery, Geneva University Hospitals, Rue Gabrielle-Perret-Gentil 4, 1211 Geneva 14, Switzerland. E-mail: jeremy.meyer@hug.ch

**Supplementary Materials - Index**

| **Supplementary Figures and Tables** |  |
| --- | --- |
| Supplementary table S1 | *pag. 2* |
| Supplementary table S2 | *pag. 3* |
| Supplementary table S3 | *pag. 4* |
| **TUBE Study Group** | *pag. 5* |
|  |  |

**Supplementary Figures and Tables**

**Supplementary Table S1**

| Characteristics | n | % |
| --- | --- | --- |
| Total respondents (complete responses) | 277 | — |
| Age, median (IQR), years | 44 (38–52) | — |
| Gender |  |  |
| Male | 225 | 81.2 |
| Female | 49 | 17.7 |
| Prefer not to say | 2 | 0.7 |
| Specialty |  |  |
| General surgery | 121 | 43.7 |
| Lower GI surgery | 98 | 35.4 |
| HPB surgery | 26 | 9.4 |
| Urology | 13 | 4.7 |
| Upper GI surgery | 12 | 4.3 |
| In training | 6 | 2.2 |
| Practice setting |  |  |
| Public academic | 152 | 54.9 |
| Public non-academic | 65 | 23.5 |
| Private | 34 | 12.3 |
| Mixed | 25 | 9.0 |
| Annual operative volume |  |  |
| 1–50 procedures | 33 | 11.9 |
| 50–100 procedures | 78 | 28.2 |
| 100–150 procedures | 62 | 22.4 |
| 150–200 procedures | 41 | 14.8 |
| >200 procedures | 60 | 21.7 |
| Country of practice (top 5) |  |  |
| Switzerland | 95 | 34.3 |
| Italy | 67 | 24.2 |
| Greece | 14 | 5.1 |
| United Kingdom | 14 | 5.1 |
| France | 10 | 3.6 |

Respondent characteristics*. IQR, interquartile range; GI, gastrointestinal; HPB, hepatopancreatobiliary.*

**Supplementary Table S2**

| Knowledge items | Correct n (%) | Not sure n (%) |
| --- | --- | --- |
| Origin of high-grade serous ovarian cancer (fallopian tube) | 102 (36.8) | 31 (11.2) |
| Effect of bilateral salpingo-oophorectomy on ovarian cancer risk (decreases risk) | 165 (59.6) | 85 (30.7) |
| Effect of opportunistic salpingectomy on ovarian cancer risk (decreases risk) | 113 (40.8) | 113 (40.8) |
| Hormonal impact: oophorectomy removes ovarian hormone production, salpingectomy does not | 202 (72.9) | 52 (18.8) |
| Long-term consequences of pre-menopausal oophorectomy (any correct item selected) | — | 50 (18.1) |
| Osteoporosis | 215 (77.6) | — |
| Cardiovascular disease | 154 (55.6) | — |
| Cognitive impairment | 87 (31.4) | — |
| Increased all-cause mortality | 64 (23.1) | — |

Knowledge of high-grade serous ovarian cancer pathogenesis and prevention

*HGSC, high-grade serous carcinoma.*

**Supplementary Table S3**

| Barrier / Facilitator items | Agree / Strongly agree n (%) | Median score (1–5) |
| --- | --- | --- |
| Would consider salpingectomy if clear abdominal surgery guidance existed | 240 (86.6) | 2 |
| Lack of clarity on consent is a barrier | 181 (65.3) | 2 |
| Lack of epidemiologic evidence in abdominal surgery populations is a barrier | 170 (61.4) | 2 |
| Adnexal prevention procedures are outside my scope of practice | 143 (51.6) | 2 |
| Concerned about added complications | 109 (39.4) | 3 |
| Access to gynecology support limits feasibility | 81 (29.2) | 3 |
| Additional operative time / complexity is a barrier | 67 (24.2) | 4 |

Barriers and facilitators to opportunistic salpingectomy during non-gynaecologic abdominal surgery

*Likert scale: 1 = strongly agree, 5 = strongly disagree. Scores 1–2 denote agreement.*

**TUBE Study Group**

**Francesco Abboretti** (Lausanne University Hospital (CHUV), Lausanne, Switzerland); **Ahmed Abdelsamad** (Department of Surgery, University of Witten/Herdecke, Witten, Germany); **Michel Adamina** (Inselspital Bern University Hospital, Bern, Switzerland); **Alberto Aiolfi** (IRCCS Ospedale Galeazzi-Sant'Ambrogio, Milan, Italy); **Vusal Aliyev** (Department of General Surgery, Alibey Hospital, Istanbul, Turkey); **Elissavet Anestiadou** (4th Department of General Surgery, General Hospital of Thessaloniki 'G. Papanikolaou', Aristotle University of Thessaloniki, Thessaloniki, Greece); **Alazar Berhe Aregawi** (Hawassa University College of Medicine and Health Sciences, Hawassa, Ethiopia); **Claudia Armellin** (Policlinico di Abano Terme, Abano Terme, Italy); **Andrea Barberis** (E.O. Ospedali Galliera, Genoa, Italy); **Daniel Benamran** (Division of Urology, Geneva University Hospitals, Geneva, Switzerland); **Stephan Bischofberger** (HOCH Kantonsspital St. Gallen, St. Gallen, Switzerland); **Luca Domenico Bonomo** (Rivoli Hospital, Rivoli (TO), Italy); **Giuseppe Brisinda** (Fondazione Policlinico Universitario A. Gemelli IRCCS, Rome, Italy); **Maria Bubenova** (Landesklinikum Waidhofen an der Ybbs, Austria, Austria); **Nicolas Buchs** (Hôpital de La Tour, Geneva, Switzerland); **Pamela Buchwald** (Skåne University Hospital Malmö, Lund University, Malmö, Sweden); **Giacomo Calini** (Department of Medical and Surgical Sciences, Alma Mater Studiorum – University of Bologna; IRCCS Azienda Ospedaliero-Universitaria di Bologna, Bologna, Italy); **Rory Callan** (St Mark's Hospital, London, United Kingdom); **Valentin Calu** (University of Medicine and Pharmacy Carol Davila, Bucharest, Romania); **Fabio Carbone** (Istituto Nazionale Tumori IRCCS – Fondazione G. Pascale, Naples, Italy); **Marco Catarci** (Sandro Pertini Hospital, Rome, Italy); **Alexandros Chamzin** (First Propaedeutic Department of Surgery, Hippocration General Hospital of Athens, Athens, Greece); **Luca Cigagna** (CHU de La Réunion, Saint-Denis, France); **Diego Coletta** (Digestive and HPB Surgery Unit, IRCCS Regina Elena National Cancer Institute, Rome, Italy); **Renan Carlo Colombari** (Gregorio Marañón University Hospital, Madrid, Spain); **Nicola Colucci** (Department of Surgery, University of British Columbia, Vancouver, Canada); **Rogier Crolla** (Netherlands, Netherlands); **Justin Davies** (Cambridge University Hospitals NHS Foundation Trust, Cambridge, United Kingdom); **Aeris Jane Dublado** (Allied Care Experts (ACE) Medical Center Tacloban, Tacloban, Philippines); **Lorenzo Epis** (DISC – Università degli Studi di Genova, Genoa, Italy); **Francesco Ferrara** (University of Palermo, Palermo, Italy); **Tahar Fillali** (Department of Surgery, Béni Messous University Hospital, Algiers, Algeria); **Christina Fleming** (University Hospital Limerick, School of Medicine, University of Limerick, Limerick, Ireland); **Gaetano Florio** (ASL Roma 5, Rome, Italy); **Pietro Fransvea** (Fondazione Policlinico Universitario A. Gemelli IRCCS, Rome, Italy); **Zoe Garoufalia** (6th Surgical Department, Hygeia Hospital, Athens, Greece); **Georgios Geropoulos** (Watford General Hospital, Watford, United Kingdom); **Eleftherios Gialamas** (Geneva University Hospitals, Geneva, Switzerland); **Guillaume Giudicelli** (Division of Visceral Surgery, Geneva University Hospitals, Geneva, Switzerland); **Ugo Giustizieri** (Ospedale Vito Fazzi, Lecce, Italy); **Carmen Loredana Gorgan** (Regina Maria Hospital, Bucharest, Romania); **Gianpiero Gravante** (Azienda Sanitaria Locale (ASL) Lecce, Lecce, Italy); **Argyrios Ioannidis** (Athens Medical Center, Athens, Greece); **Orestis Ioannidis** (4th Department of Surgery, Medical School, Aristotle University of Thessaloniki, Thessaloniki, Greece); **Arda Isik** (Istanbul Medeniyet University, Istanbul, Turkey); **Ioannis Katsaros** (National and Kapodistrian University of Athens, Laikon General Hospital, Athens, Greece); **Deborah Keller** (Mayo Clinic, Phoenix, AZ, United States); **Raja Basit N. Khan** (Airedale NHS Foundation Trust, Keighley, United Kingdom); **Niels Komen** (University Hospital Antwerp, Antwerp, Belgium); **Dimitris Korkolis** (Department of Surgery, General Anticancer Oncology Hospital of Athens 'Saint Savvas', Athens, Greece); **Bojan Kovacevic** (UMC Zvezdara, Belgrade, Serbia); **Joël L. Lavanchy** (Clarunis – University Digestive Health Care Center, Basel, Switzerland); **Jeroen Leijtens** (Laurentius Ziekenhuis, Roermond, Netherlands); **Emilie Liot** (Geneva University Hospitals, Geneva, Switzerland); **Alexis Litchinko** (Department of Surgery, Bordeaux Colorectal Academy, Bordeaux, France); **Andrea Pierre Luzzi** (ASST Santi Paolo e Carlo, Milan, Italy); **Jin Jiun Mah** (Queen Elizabeth Hospital, Kota Kinabalu, Malaysia); **Kashish Malhotra** (Department of Surgery, Rama Medical College Hospital and Research Centre, Kanpur, India); **Draga-Maria Mandi** (Coltea Clinical Hospital, Bucharest, Romania); **Angelo Alessandro Marra** (Isola Tiberina – Gemelli Isola Hospital, Rome, Italy); **Valentina Miacci** (Campus Bio-Medico, Rome, Italy); **Pamela Milito** (IRCCS Policlinico San Donato, San Donato Milanese, Italy); **Ana Minaya Bravo** (Hospital Universitario del Henares, Madrid, Spain); **Blanca Montcusí** (Section of Colon and Rectal Surgery, Department of Surgery, Hospital del Mar, Barcelona, Spain); **Dimitrios Moris** (Department of Surgery, MedStar Georgetown University Hospital, Washington, DC, United States); **Adam Mylonakis** (First Department of Surgery, National and Kapodistrian University of Athens, Laiko General Hospital, Athens, Greece); **Peter Neary** (University Hospital Waterford, University College Cork, Cork, Ireland); **Harish Neelamraju Lakshmi** (Department of Surgical Oncology, Continental Hospitals, Hyderabad, India); **Ionut Negoi** (Carol Davila University of Medicine and Pharmacy; Clinical Emergency Hospital of Bucharest, Bucharest, Romania); **Antonella Nicotera** (Rivoli Hospital ASL TO3, Rivoli, Italy); **Aristotelis Nikitaras** (Athens Euroclinic, Athens, Greece); **Nuri Okkabaz** (Istanbul Atlas University, Istanbul, Turkey); **Lorenzo A. Orci** (Clinique Générale-Beaulieu, Geneva, Switzerland); **Paolo Ossola** (General Surgery 2U – Liver Transplant Centre, A.O.U. Città della Salute e della Scienza, Turin, Italy); **Livia Palmieri** (Ospedale Madre Giuseppina Vannini, Rome, Italy); **Giulia Paradiso** (Ospedale M.G. Vannini, Rome, Italy); **Francesco Pata** (Department of Pharmacy, Health and Nutritional Sciences, University of Calabria, Rende, Italy); **Georgios Peros** (Chirurgie Zentrum St. Anna, Lucerne, Switzerland); **Lorenzo Petagna** (AOU Pisana, Pisa, Italy); **Thalia Petropoulou** (Aretaieio University Hospital, Athens, Greece); **Gopi Ramu** (Department of Surgery, Chengelpet Medical College and Hospital, Chengelpet, India); **Stefano Restaino** (Azienda Sanitaria Universitaria Friuli Centrale, Udine, Italy); **Raffaello Roesel** (EOC Regional Hospital of Lugano, Lugano, Switzerland); **Fausto Rosa** (Department of Surgery, Università Cattolica del Sacro Cuore; Fondazione Policlinico Universitario Agostino Gemelli IRCCS, Rome, Italy); **Fabian Rössler** (Department of Surgery and Transplantation, University Hospital Zurich, Zurich, Switzerland); **Jacques-Emmanuel Saadoun** (Department of Surgical Oncology, Institut Paoli-Calmettes, Marseille, France); **Kapil Sahnan** (St Mark's Hospital, London, United Kingdom); **Ville Sallinen** (Helsinki University Hospital and University of Helsinki, Helsinki, Finland); **Matteo Santoliquido** (IRCCS Ospedale Policlinico San Martino, Genoa, Italy); **Giulio Aniello Santoro** (Humanitas Gavazzeni Hospital, Bergamo, Italy); **Carlo Alberto Schena** (Fondazione Poliambulanza Istituto Ospedaliero, Brescia, Italy); **Boris Schiltz** (Centre Hospitalier de Bienne, Biel/Bienne, Switzerland); **Dimitrios Schizas** (First Department of Surgery, National and Kapodistrian University of Athens, Athens, Greece); **Michele Scopelliti** (Chirurgia 2, ULSS2 Marca Trevigiana, Treviso, Italy); **Mostafa Shalaby** (Department of General Surgery, Mansoura University, Mansoura, Egypt); **Marwan-Julien Sleiman** (Geneva University Hospitals, Geneva, Switzerland); **Amine Souadka** (Department of Surgical Oncology, National Institute of Oncology, Mohammed V University, Rabat, Morocco); **Moritz Benjamin Sparn** (HOCH Health Ostschweiz Kantonsspital St. Gallen, St. Gallen, Switzerland); **George Stavrou** (417 NIMTS Hospital, Athens, Greece); **Giovanni Tebala** (Department of Digestive and Emergency Surgery, Azienda Ospedaliera S. Maria, Terni, Italy); **Pierre-Alain Tokoto** (Geneva University Hospitals, Geneva, Switzerland); **Giovanni Tomasicchio** (Azienda Consorziale Policlinico di Bari, Bari, Italy); **Charles Van Praet** (Department of Urology, Ghent University Hospital, Ghent, Belgium); **Gabrielle H. van Ramshorst** (Ghent University Hospital, Ghent University, Ghent, Belgium); **Carsten T. Viehl** (Centre Hospitalier de Bienne, Biel/Bienne, Switzerland); **Danilo Vinci** (Hospital del Mar, Barcelona, Spain); **Giuseppe Vizzielli** (University of Udine, Udine, Italy); **Lukas Werner Widmer** (Department of Surgery, Cantonal Hospital of Fribourg; University of Fribourg, Fribourg, Switzerland); **Muhammad Umar Younis** (Mediclinic City Hospital Dubai, Dubai, United Arab Emirates); **Matteo Zanello** (Department of General Surgery, IRCCS Azienda Ospedaliero-Universitaria di Bologna, Maggiore Hospital, Bologna, Italy).
